# Biochemistry and regulation of histone lysine l-lactylation

**DOI:** 10.1038/s41580-025-00876-7

**Published:** 2025-08-19

**Authors:** Xinlei Sheng, Hening Lin, Philip A. Cole, Yingming Zhao

**Affiliations:** 1 The Ben May Department for Cancer Research, The University of Chicago, Chicago, IL, USA.; 2 Howard Hughes Medical Institute, Department of Medicine, The University of Chicago, Chicago, IL, USA.; 3 Howard Hughes Medical Institute, Department of Chemistry, The University of Chicago, Chicago, IL, USA.; 4 Division of Genetics, Department of Medicine, Brigham and Women’s Hospital, Boston, MA, USA.; 5 Department of Biological Chemistry and Molecular Pharmacology, Harvard Medical School, Boston, MA, USA.

## Abstract

Histone l-lactylation is a newly identified, metabolism-linked short-chain Lys acylation. Mounting evidence indicates that Lys l-lactylation has key roles in transcription regulation and many other cellular processes and is associated with diverse pathophysiological changes. In this Review, we discuss the unique features of histone l-lactylation, emphasizing the differences between l-lactylation and its isomers, such as d-lactylation. We discuss the regulation of l-lactylation by writers and erasers, its readers and its cofactor l-lactyl-CoA. We highlight the dynamic regulation of nuclear l-lactyl-CoA and l-lactyl-CoA synthetases, which are crucial determinants of the specificity of histone Lys l-lactylation. We also discuss an emerging l-lactyl-CoA-independent l-lactylation pathway. By integrating these findings, we aim to deepen our understanding of the biochemistry and regulation of histone l-lactylation and its broad biological significance.

## Introduction

**Fig. 1 |:**
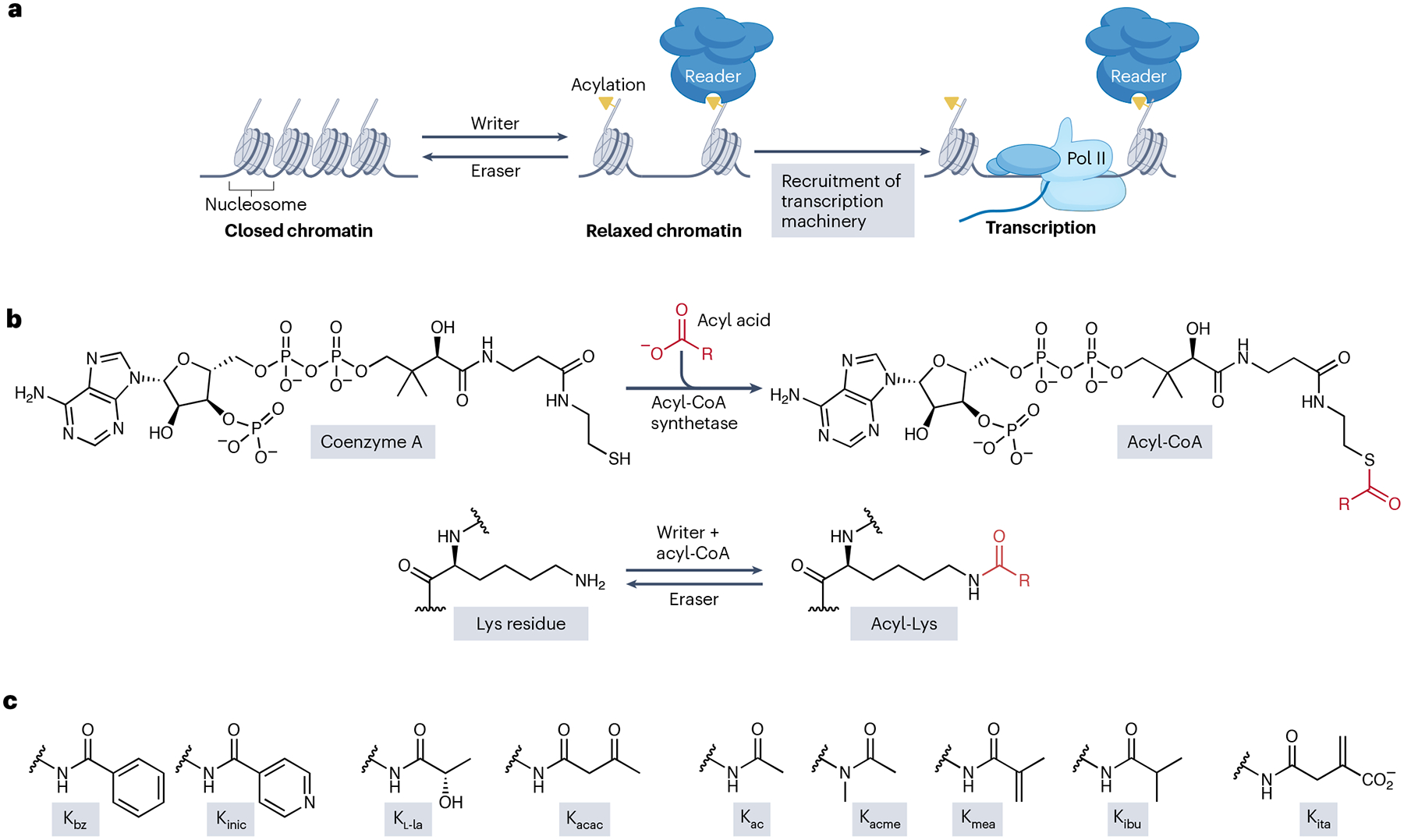
Overview of histone Lys acylation and of recently identified Lys acylations. **a**, A schematic representation of Lys acylation-mediated chromatin regulation. The writer enzymes add acyl groups and eraser enzymes remove them, thereby dynamically modulating chromatin accessibility by facilitating the transition between closed and relaxed chromatin. The reader proteins regulate gene transcription by recognizing the acylated histones and recruiting the transcription machinery. **b**, Top: in acyl-CoA synthesis, acyl acid is converted to acyl-CoA by acyl-CoA synthetase. R represents the specific acyl group. Bottom: acyl-CoA is then used by specific writer proteins to catalyse the corresponding acylation reaction. The wavelets represent the connections of the modified Lys residues to the protein backbone. **c**, The chemical structures of recently discovered acylations of histone Lys residues, categorized into four distinct groups. The wavelets represent connections of the post-translational modifications to the modified Lys residues. K_ac_, Lys acetylation; K_acac_, Lys acetoacetylation; K_acme_, Lys acetyl-methylation; K_bz_, Lys benzoylation; K_ibu_, Lys isobutyrylation; K_inic_, Lys isonicotinylation; K_ita_, Lys itaconylation; K_l-la_, Lys l-lactylation; K_mea_, Lys methacrylation; Pol II, RNA polymerase II.

Histone proteins, the fundamental components of chromatin, undergo a variety of post-translational modifications (PTMs)^1–3^. Histone PTMs are crucial for regulating chromatin structure and function by altering chromatin compaction, histone exchange and turnover, and the recruitment of effector proteins^4–8^. Specifically, these modifications have a pivotal role in regulating chromatin state — shifting between open (transcriptionally active) and condensed (transcriptionally repressive) conformations (Fig. 1a). It is therefore not surprising that the dysregulation of these PTMs has been linked to many diseases, including cancer, diabetes and neurological disorders^9–15^.

Histone PTMs are dynamically regulated by ‘writer’ and ‘eraser’ enzymes, which add and remove them, respectively^16^. In addition, ‘reader’ proteins recognize specific histone PTMs and integrate them into signalling networks by recruiting transcriptional cofactors^16^, chromatin remodellers and other regulatory proteins (Fig. 1a,b). This regulatory system of writers, erasers and readers ensures that histone modifications can fine-tune gene expression programs tailored to the metabolic and cellular environment.

Transcriptional regulation is a key mechanism by which cells adapt to metabolic changes^2,17^. Over the past two decades, extensive studies have revealed a close link between cellular metabolism and histone modifications^18,19^. Classic examples include histone Lys acetylation (K_ac_) and Lys methylation, which are regulated by a variety of cellular metabolites, including acetate, citrate, acetyl-CoA, *S*-adenosylmethionine, α-ketoglutarate, nicotinamide adenine dinucleotide (NAD^+^) and ATP^20–23^. These modifications also serve as sensors of external stimuli. For example, hypoxia, oxidative stress or inflammatory signals can modulate PTMs by altering the activity of metabolic enzymes, compartmentalized metabolite synthesis or post-translational regulation of writers and erasers.

Recent advances, particularly the use of high-sensitivity mass spectrometry, alongside complementary verification methods, have driven the identification of additional histone Lys short-chain lipid acylations^3,24^. Although these newly discovered acylations share similarities with the well-studied K_ac_ (Fig. 1c), their biochemical features enable distinct modulations of chromatin structure and gene regulation^3,25–27^. Among these PTMs, Lys l-lactylation (K_l-la_) — the addition of an l-lactyl group onto the ε-amine of a Lys amino acid residue — has garnered considerable attention due to its close association with l-lactate, its precursor, which is associated with a variety of physiopathological conditions, for example, with hypoxia and the Warburg effect^28,29^. Since its discovery, K_l-la_ has been associated with a wide range of cellular and physiopathological processes, such as neural activity, tumour progression, immune responses, cell pluripotency and DNA repair^28,30–38^.

In this Review, we discuss the chemistry and biochemistry of K_l-la_. We begin by discussing the distinct metabolic pathways driving the formation of lactylation isomers, with a focus on the methodologies used for their analysis. We then discuss the regulation of K_l-la_, including its addition, removal and interactions with other proteins. Furthermore, we discuss the synthesis and regulation of l-lactyl-CoA, the precursor of K_l-la_. Finally, we discuss an emerging l-lactyl-CoA-independent K_l-la_ pathway and compare it with the established l-lactyl-CoA-dependent K_l-la_ pathway. The functions of histone K_l-la_ are beyond the scope of this Review; we encourage readers seeking more information on this aspect of K_l-la_ to consult other articles^25–27,39–44^. For more information on the identification and biochemical investigation of other short-chain lipid Lys acylations, we recommend the original research papers^28,45–51^.

## Overview of recently discovered histone Lys acylations

Since our previous review of histone Lys acylations^3^, eight new types of histone Lys acylation have been identified (Supplementary Table 1), substantially expanding the diversity of histone PTMs^28,45–51^. These newly described histone acylations are bulkier than the extensively studied K_ac_ and Lys methylation and have unique functional groups with the potential to create a more open and accessible chromatin state (Fig. 1a,c). For instance, Lys methacrylation incorporates a methacrylyl group containing a double bond^49^, which increases the rigidity of the side chain, thereby restricting conformational flexibility around the modified Lys residue. Lys benzoylation introduces a benzoyl group, thereby adding a bulky aromatic ring to the Lys residue^45^. The ring structure provides a planar conformation capable of engaging in π–π stacking interactions with aromatic amino acids in chromatin-associated proteins. Similarly, Lys isonicotinylation adds a bulky pyridine ring^46^, but the nitrogen atom in the pyridine ring introduces a lone pair of electrons, leading to an uneven electron distribution. This difference in electron distribution makes pyridine more polar and basic than the ‘standard’ benzene ring, thereby enabling dipole–dipole interactions with other molecules. Lys isobutyrylation (K_ibu_) differs from the previously identified Lys butyrylation (K_bu_), its constitutional isomer, by its branched carbon chain^50^. Lys acetoacetylation features a longer chain than K_ac_, and it can be stimulated by acetoacetate, a major ketone body^47^; Lys acetyl-methylation has both an acetyl group and a methyl group added to the Lys ε-amino group^48^.

**Fig. 2 |:**
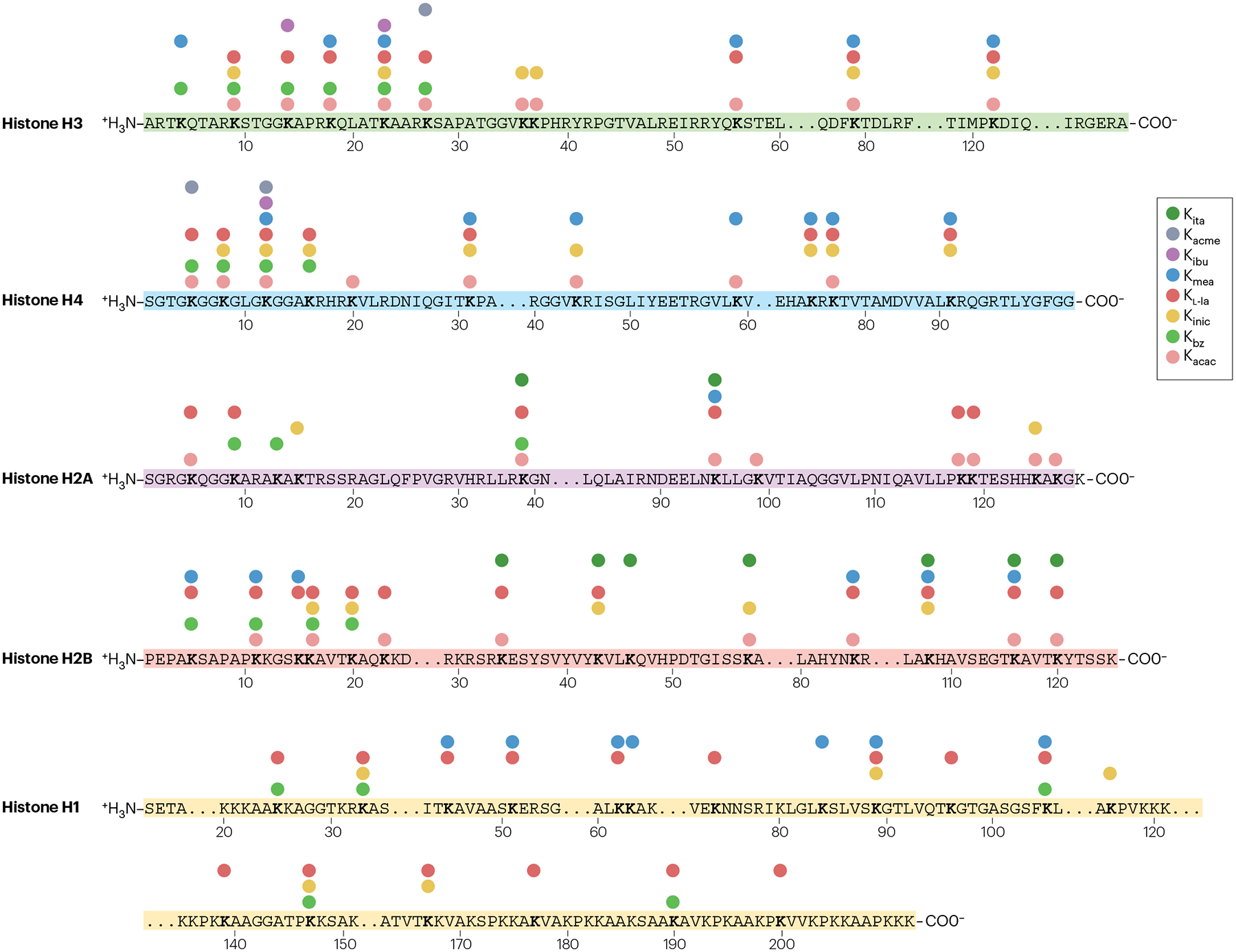
Distribution map of recently identified acyl-Lys histone modifications. The amino acid sequences of the histone proteins are shown with the modified Lys residues in bold. K_acac_, Lys acetoacetylation; K_acme_, Lys acetyl-methylation; K_bz_, Lys benzoylation; K_ibu_, Lys isobutyrylation; K_inic_, Lys isonicotinylation; K_ita_, Lys itaconylation; K_l-la_, Lys l-lactylation; K_mea_, Lys methacrylation. For comparison, the landscape of Lys acetylation (K_ac_) can be found in previous reviews^3,204,205^.

These histone acylations broaden the chemical landscape of histone PTMs (Fig. 2) by modifying key Lys residues such as H3K9, H3K18, H3K27, H3K56, H4K5, H4K8 and H4K16 — residues known to regulate transcription. Because these PTMs differ in charge, hydrophobicity and steric bulk, distinct acylations at the same site may exert different effects on transcription regulation. Other mapped sites, though less characterized, may also influence chromatin architecture and effector binding. Among these acylations, K_l-la_ emerges as the most studied, given its connections to glycolysis, hypoxia and many physiopathological changes^28,39,42,43^. Derived from l-lactate, K_l-la_ involves the addition of an l-lactyl group to a histone Lys residue. The l-lactyl group, which is bulkier than K_ac_, contains a hydroxyl group capable of forming hydrogen bonds with other proteins, thereby affecting chromatin structure and function^28^.

The expanded chemical diversity of the new Lys acylations allows for finer regulation of chromatin structure and accessibility. Their precursors, short-chain lipids, can stimulate Lys acylations, suggesting that metabolism-coupled epigenetic changes enable rapid adaptation of gene expression to cellular changes.

## The three Lys lactylation isomers

Identifying a PTM by mass spectrometry-based techniques involves determining its elemental composition from the observed mass shift of a peptide of interest^3,24^. However, the same mass shift at an amino acid of interest can be ascribed to multiple isomers. Isomers are molecules that differ in atomic arrangement but share the same molecular formula^52^. Constitutional isomers differ in the connectivity of their atoms, whereas stereoisomers share the same connectivity but differ in the spatial arrangement of atoms around a chiral centre. Given their structural differences, different PTM isomers may interact with different sets of proteins, including writers, erasers and readers. Notable examples of PTM isomers include K_bu_ and K_ibu_, and K_l-la_ and Lys d-lactylation (K_d-la_). K_bu_ and K_ibu_ are constitutional isomers, with K_bu_ having a straight-chain butyryl group and K_ibu_ having a branched-chain isobutyryl group^50^. K_l-la_ and K_d-la_ are stereoisomers, characterized by distinct three-dimensional arrangement around the chiral centre of the lactyl group. Despite their structural similarity, these stereoisomers are formed in distinct metabolic pathways, exemplifying the intricate interplay between cellular metabolism and histone modifications.

**Fig. 3 |:**
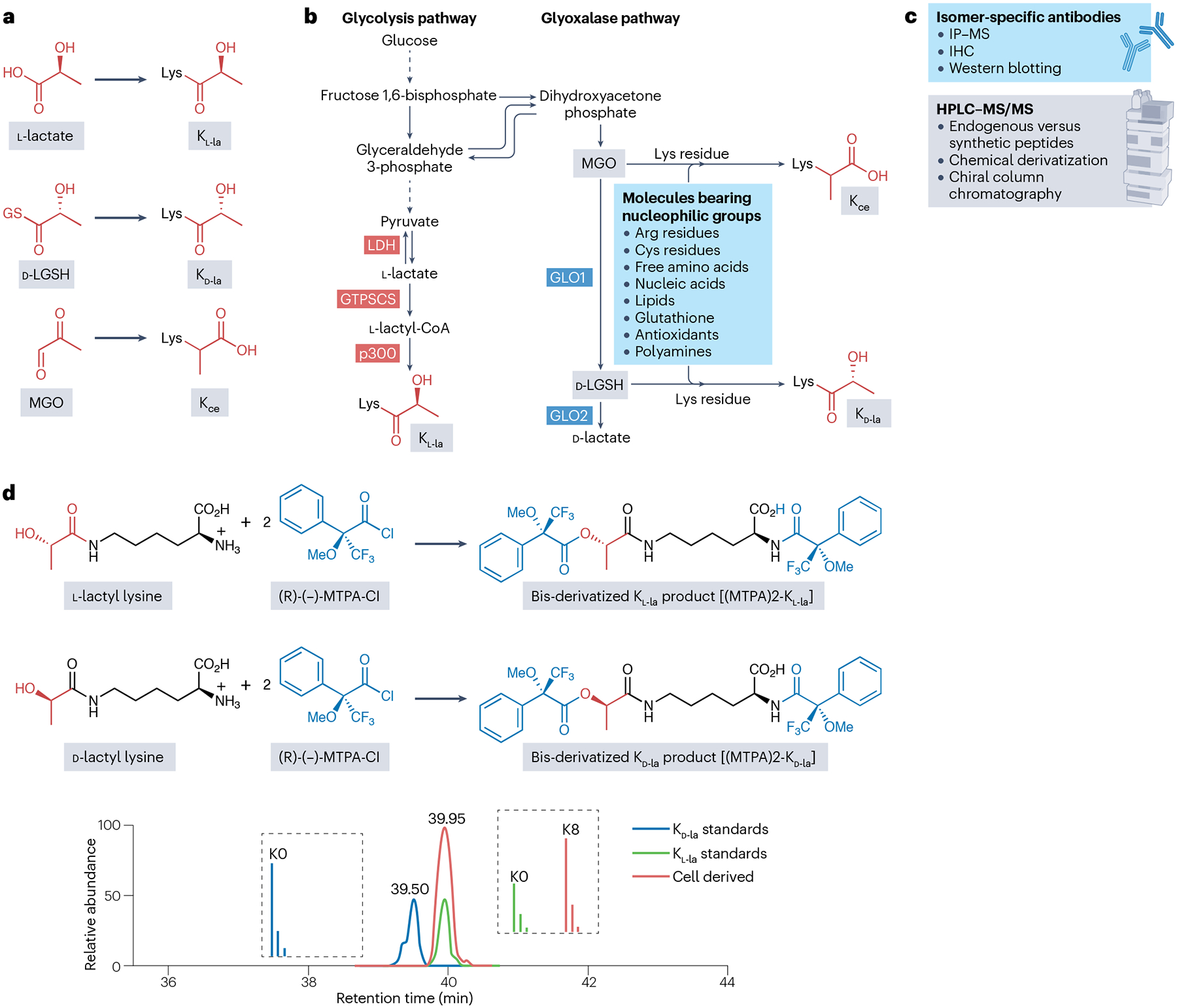
The Lys lactylation isomers and how to distinguish between them. **a**, The three Lys lactylation isomers include l-lactylation (K_l-la_), which is derived from l-lactate, d-lactylation (K_d-la_), which is derived from *S*-d-lactoylglutathione (LGSH), and *N*-ε-(carboxyethyl)-lysine (K_ce_), which is derived from methylglyoxal (MGO). **b**, The metabolic pathways involved in generation of K_l-la_, K_d-la_ and K_ce_. The key enzymes and potential side reactions are indicated. In addition to Lys residues, MGO and LGSH are also highly reactive with a broad range of nucleophilic molecules (blue box), including Arg and Cys residues, nucleic acids and lipids. **c**, Analytical methods for detecting and characterizing Lys lactylation isomers, featuring two main approaches: immunological methods using isomer-specific antibodies, including immunoprecipitation coupled with mass spectrometry (IP–MS), immunohistochemistry (IHC) and western blotting, and chromatography-based methods using high-performance liquid chromatography coupled with tandem mass spectrometry (HPLC–MS/MS) analysis with chiral separation capabilities. This method can compare endogenous peptides with synthetic peptide standards to distinguish constitutional isomers. Chemical derivatization using Mosher’s reagent and chiral column, when combined with HPLC–MS/MS, allows for stereochemical discrimination between K_l-la_ and K_d-la_. **d**, The chemical derivatization reactions and chromatography separation of Lys lactylation isomers. Top: the chemical derivatization reactions between l-lactyl Lys or d-lactyl Lys and methoxy-α-(trifluoromethyl)phenylacetyl chloride (MTPA-Cl; Mosher’s acid chloride). This reaction yields bis-derivatized products that amplify the stereochemical differences between the two isomers. Bottom: the extracted ion chromatograms from HPLC–MS/MS show the separation of the chemically derivatized synthetic K_l-la_ and K_d-la_ amino acid standards (light, K0) and the lactylated amino acids derived from cellular peptides (heavy labelled, K8). The extracted ion chromatograms demonstrate the presence of K_l-la_ in cell-derived peptide. GLO1, glyoxalase 1; GTPSCS, GTP-specific succinyl-CoA synthetase; LDH, lactate dehydrogenase. Panel **d** adapted from ref. 53, Springer Nature Limited.

Three Lys lactylation isomers have been reported: K_l-la_, K_d-la_ and *N*-ε-(carboxyethyl)-lysine (K_ce_)^53,54^ (Fig. 3a). The prevalence of K_l-la_ was supported by diverse lines of evidence from a previous study^28^ and subsequently confirmed by hundreds of studies using l-lactyllysine antibodies in diverse biological systems^55–67^. Moreover, the identification of lactyltransferases and their role in regulating K_l-la_ provide strong evidence that K_l-la_, rather than K_d-la_, is dynamically regulated in various cellular conditions, including but not limited to glycolysis and oncogenic signalling^28,53,65,68,69^.

In contrast to the enzymatically regulated K_l-la_, K_ce_ and K_d-la_ are formed non-enzymatically (Fig. 3b). Although K_ce_ has long been reported^70^, its biological functions remain largely uncharacterized. K_ce_ is derived from the reaction of methylglyoxal (a glycolysis by-product) with Lys residues. Methylglyoxal is highly reactive and toxic and is maintained at low levels by the glyoxalase system to mitigate cellular damage^52^. It can be converted into *S*-d-lactoylglutathione (d-lactyl-GSH or LGSH) by glyoxalase 1 (GLO1); in turn, LGSH modifies Lys residues to form K_d-la_ in a non-enzymatic manner^54,71^. Thus, whereas K_l-la_ is dynamically regulated by enzymes, the existing literature suggests that K_ce_ and K_d-la_ are formed through non-enzymatic reactions with reactive metabolic byproducts.

### Detecting Lys lactylation isomers and studying their dynamics

We reason that two structurally identical molecules bearing a PTM, either endogenous or chemically synthesized, should exhibit identical chemical properties in analytical assessments. Constitutional isomers can usually be separated by reverse-phase high-performance liquid chromatography (HPLC), such as Lys crotonylation (K_cr_) isomers^72^ and Lys 3-hydroxybutyrylation (K_bhb_) isomers^73^. However, stereoisomers are generally difficult to resolve with HPLC, posing a challenge for current analytical methods^53^. Although HPLC separation with chiral beads or a nuclear magnetic resonance method can, in principle, distinguish between stereoisomers (Fig. 3c), they lack sensitivity for analysis of histone peptides. To overcome this challenge, two orthogonal methods have been developed and used to differentiate stereoisomers of K_l-la_ and determine their abundance^53,74^. K_ce_ can be easily separated from its constitutional isomers, either K_l-la_ or K_d-la_, by reverse-phase HPLC^53^. To further resolve K_l-la_ and K_d-la_, PTM-isomer-specific antibodies were developed, including pan-K_ce_, pan-K_l-la_ and pan-K_d-la_ antibodies (Fig. 3c). These antibodies, raised against peptides (antigens) bearing specific lactylation isomers of interest, can differentiate the isomers on peptide and protein levels with comparable binding affinity^53,74^. Another orthogonal method is modified single amino acid analysis, in which peptide digestion into single amino acids is followed by chemical derivatization with Mosher’s acid chloride, a reagent commonly used to differentiate chiral molecules and determine their stereo-configurations^53^. This derivatization reaction generates bis-derivatized products — (MTPA)_2_-K_l-la_ or (MTPA)_2_-K_d-la_ — thus amplifying the chiral difference between K_l-la_ and K_d-la_ (Fig. 3d). The resulting modified K_l-la_ and K_d-la_ amino acid derivatives can then be easily separated in reverse-phase HPLC (Fig. 3d). The combination of such two orthogonal methods allows us to distinguish between and study the dynamics of the three isomers under various biological conditions^53,74^.

The two orthogonal methods were used to investigate the structures and dynamic changes of the three lactylation isomers^53,74^. The three antibodies targeting these isomers were used to enrich the corresponding PTM peptides of interest, followed by peptide identification using HPLC with tandem mass spectrometry^53,74^. A total of 166 K_l-la_-modified histone peptides were identified in various conditions, including high glucose, oncogenic signalling and hypoxia^53,74^. By sharp contrast, neither K_d-la_-containing nor K_ce_-containing peptides were detectable. Western blotting analysis using the K_ce_ and K_d-la_ antibodies further confirmed that the signals of these two PTMs were at background levels in the tested conditions^53,74^. Modified single amino acid analysis was used to measure the three modifications at the single amino acid level, showing that whereas K_l-la_ could be easily detected, K_d-la_ and K_ce_ were not observed under the same conditions^53^. Taken together, these results clearly indicate that K_d-la_ and K_ce_ are either absent or present at extremely low levels in the tested cells and conditions^53,74^. We conclude that K_l-la_ is the predominant isomer of lactylation that is induced in conditions of high glucose, oncogenic signalling and hypoxia^53,74^.

### The basis of low abundance of K_d-la_ and K_ce_

The predominance of K_l-la_ in the aforementioned conditions is consistent with several lines of evidence: (1) glycolysis-derived lactate is in l-form^29,75^; (2) mammalian glycolytic lactate dehydrogenases (LDHA and LDHB) produce l-lactate rather than d-lactate^76,77^; (3) exogenous l-lactate but not d-lactate stimulates K_l-la_^28,38,39,78^; (4) K_l-la_ can be detected by the isomer-specific l-lactyllysine antibodies in numerous studies^28,34,36–39,53,56,65,67,74,79–85^; (5) l-lactyl-CoA serves as a substrate of Lys acetyltransferase (KATs) such as p300, CREB-binding protein (CBP), KAT5, KAT8, KAT7 and KAT2A, which catalyse histone K_l-la_ and regulate transcription^28,34,38,53,65,67,68^; (6) isotopically labelled glucose and l-lactate can be incorporated into K_l-la_^28,79^; (7) isotopically labelled l-lactate can be incorporated into l-lactyl-CoA^53,79^; and (8) chromatin immunoprecipitation sequencing (ChIP–seq) experiments suggest a nonrandom distribution of histone K_l-la_ in chromosomes, providing evidence against non-enzymatic histone lactylation^28,35,39,86^.

The low abundances of K_ce_ and K_d-la_ probably arise from low concentrations of their precursors, methylglyoxal and LGSH, respectively. To alleviate its cellular toxicity, methylglyoxal in mammals is kept in low extracellular and intracellular concentrations of <1 and 10 μM, respectively^71,87,88^. LGSH is generated by reaction of methylglyoxal with glutathione (to form a hemithioacetal intermediate), followed by isomerization by GLO1 (ref. 71) (Fig. 3b). LGSH is further converted to d-lactate by GLO2, keeping its plasma concentration low (<30 μM in both healthy individuals and individuals with diabetes^89,90^). The low concentrations and stringent regulation of these precursors keep K_ce_ and K_d-la_ at very low levels. By contrast, the intracellular and plasma l-lactate levels are typically in the submillimolar range and can rise up to 20–40 mM under physiopathological conditions^29,91–96^. Thus, the concentration of l-lactate is typically 100–10,000 times higher than those of methylglyoxal, LGSH and d-lactate^71,87–90,97^. In principle, d-lactate could be converted to d-lactyl-CoA by a hypothetical d-lactyl-CoA synthetase, assuming its existence in cells. However, d-lactate is present at very low concentrations (<20 μM)^97^. Although d-lactate is reported to be produced by lactobacilli bacteria and bifidobacteria in the gastrointestinal tract^98^, the enzyme producing d-lactyl-CoA is yet to be identified. Given the fairly low concentration of d-lactate, the formation of d-lactyl-CoA and its potential contribution to K_d-la_ formation are probably negligible.

The broad reactivity of methylglyoxal and LGSH further contributes to the low abundances of K_ce_ and K_d-la_. Methylglyoxal is a highly reactive compound that can interact with diverse molecules, including but not limited to reactive oxygen species, DNA, RNA, lipids, free amino acids and multiple amino acid residues in proteins^99–101^. In addition to K_ce_, methylglyoxal-induced protein modifications give rise to *N*-ε-(carboxymethyl)-l-lysine, argpyrimidine, hydroimidazolone derivatives, tetrahydropyrimidine and various methylglyoxal-derived crosslinked dimers^101–103^. Likewise, LGSH contains a highly reactive thioester bond, which can undergo hydrolysis or nucleophilic attack by molecules containing thiol, hydroxyl, amine or other nucleophilic groups. Thus, K_d-la_ and K_ce_ represent only two of many products of the reactions of LGSH and methylglyoxal with other molecules.

In summary, low levels of methylglyoxal and LGSH limit the production of K_ce_ and K_d-la_. Without dedicated enzymes to promote these PTMs, the broad reactivity of both compounds further reduces the likelihood of non-enzymatic K_d-la_ and K_ce_ formation. It is important to note that LGSH-induced d-lactylation has been frequently reported without specifying the stereochemistry, causing confusion in the research community. Indeed, LGSH cannot serve as a cofactor for the generation of K_l-la_. Thus, LGSH-generated lactylation is present in K_d-la_ but not K_l-la_ form. Moreover, K_d-la_ was claimed to be detected and altered in response to changes in cellular environments. In some of those studies, the K_l-la_ antibodies were used to detect LGSH-generated lactylation or K_d-la_. Clear specification of lactylation isomer (for example, K_l-la_ or K_d-la_), precursor of modification (for example, l-lactate or LGSH), and the antibodies used for detection would help prevent such confusion.

## Enzymatic regulation of histone K_l-la_

Histone K_l-la_ is regulated by multiple factors, including by lactyltransferases, delactylases and l-lactyl-lysine binding proteins, as well as by the cofactor l-lactyl-CoA and by l-lactyl-CoA metabolic enzymes.

### Addition of K_l-la_ by lactyltransferases

Histone acetyltransferases (HATs), traditionally classified as acetyltransferases, have recently been found to catalyse a wide variety of short-chain Lys acylation reactions. There are three major families of HATs: GNAT (including KAT2A, KAT2B and HAT1), MYST (KAT6A, KAT8, KAT5 and KAT7) and p300–CBP^21,26,104–107^. Each family exhibits specificities (and distinct efficiencies) for various acyl-CoA cofactors.

**Fig. 4 |:**
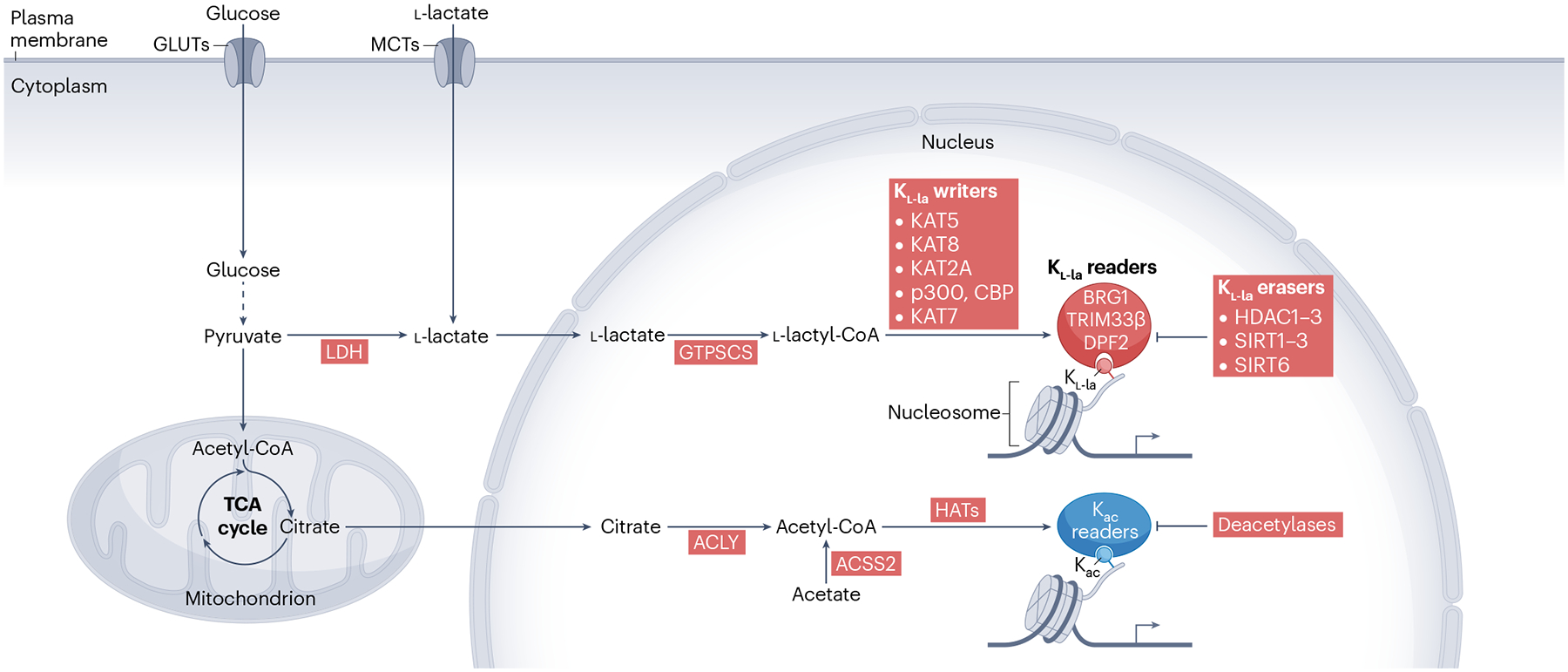
Cellular metabolic pathways and the regulation of histone K_l-la_. Glucose enters the cytoplasm through transmembrane glucose transporters (GLUTs) and undergoes glycolysis to produce pyruvate, which can either enter the tricarboxylic acid (TCA) cycle in the mitochondria to generate acetyl-CoA or be converted to l-lactate by lactate dehydrogenases (LDH). l-lactate can also enter the cell through monocarboxylate transporters (MCTs). In the nucleus, GTP-specific succinyl-CoA synthetase (GTPSCS) converts l-lactate to l-lactyl-CoA, which is then used by the indicated writers to transfer the l-lactyl group to Lys residues of histone proteins. ATP-citrate synthase (ACLY) can convert citrate to acetyl-CoA, and acetyl-CoA synthetase 2 (ACSS2) can convert acetate to acetyl-CoA; both sources of acetyl-CoA can be used for histone Lys acetylation (K_ac_). Histone Lys l-lactylation (K_l-la_) and K_ac_ can be recognized by their respective reader proteins to mediate downstream effects. Histone K_l-la_ can be removed by the indicated erasers. CBP, CREB-binding protein; HDAC, histone deacetylase; HAT, histone acetyltransferase; KAT, lysine acetyltransferase; SIRT, sirtuin.

The p300 and CBP proteins, often studied together due to their similar structures and functions, possess a deep aliphatic pocket within the active site^107^. This structural feature allows them to accommodate multiple short-chain acyl-CoAs^108^. Specifically, both p300 and CBP can catalyse the addition of K_l-la_, in agreement with their promiscuous substrate activity^28,34,53,78,84,109–111^. Among the MYST enzymes, KAT7, KAT5 and KAT8 exhibit strong K_l-la_ activity^38,65,67^. In addition, a member of the GNAT family, KAT2A, can catalyse histone K_l-la_^68^ (Fig. 4).

### Removal of K_l-la_ by delactylases

Mounting evidence shows that two types of Lys deacetylase, zinc-dependent histone deacetylases (HDACs) and NAD^+^-dependent sirtuins (SIRTs), can catalyse the removal of acyl groups from acyl-Lys residues^112–118^. Class I HDACs (HDAC1–3) have distinct roles in regulating histone K_l-la_ levels, with HDAC1 and HDAC3 regulating overall K_l-la_ levels in various cell lines and HDAC2 targeting lactylated histone H3 Lys 18 (H3K18la) and H3K9la in pancreatic ductal adenocarcinoma cells and endothelial cells, respectively^119–122^. In particular, the MiDAC and RERE HDAC1 complexes competently delactylate nucleosomal substrates, and the CoREST HDAC1 complex cleaves K_l-la_^123^. Class III HDACs (SIRT1-3) also demonstrate delactylation activity: SIRT1 and SIRT3 exhibit robust delactylase activity towards diverse substrates and modulate glycolysis by delactylating glycolysis enzymes^81,83,124^; SIRT2 is a strong delactylase both in vitro and in neuroblastoma cells^86,125^. In two recent studies, SIRT6 was reported to possess efficient delactylase activity towards H3K9la on in vitro-reconstituted nucleosomes^126,127^ (Fig. 4). Together, these findings highlight that K_l-la_ is dynamically regulated by a combination of HDACs and sirtuins, each with distinct substrate specificities.

It is worth noting that the activity of K_l-la_ erasers is probably context dependent, as their enzymatic activities can be influenced by their expression levels, subcellular localization, post-translational modifications and the availability of cofactor and precursors^128^.

### K_l-la_ readers regulate transcription

Whereas advances have been made in characterizing the writers and erasers of histone K_l-la_, the downstream effects mediated by potential readers remain elusive. Traditional classes of K_ac_ reader proteins include bromodomain-containing proteins (bromodomain-containing protein 2 (BRD2), BRD4 and TRIM33β), PHD finger domain-containing proteins (BPTF, DPF2 and ING) and YEATS domain-containing proteins (YEATS2, AF9, ENL and GAS41)^21,129^. Given the similarities between K_ac_ and K_l-la_, it is likely that some of the canonical K_ac_ readers may also serve as K_l-la_ readers. Indeed, BRG1, a bromodomain-containing protein and a member of the SWI/SNF family of chromatin remodellers, can bind to H3K18la and remodel chromatin structure during early reprogramming of induced pluripotent stem cells^130^. In addition, TRIM33β was recently reported to be a reader of H3K18la^131^, and DPF2 was identified as a reader of H3K14la^132^ (Fig. 4). The interaction between DPF2 and H3K14la is crucial for regulating chromatin remodelling and gene expression in cancer, as it promotes the expression of genes that enhance cell survival and contribute to tumorigenesis^132^.

## Regulation of histone K_l-la_ through nuclear synthesis of l-lactyl-CoA

The supply and accessibility of acyl-CoAs are crucial for the regulation of the abundance and specificity of acylations. Acetyl-CoA is a highly dynamic metabolite and can be generated in the cytosol, nucleus and mitochondria by diverse enzymes^133–137^. Nevertheless, the synthesis, abundance and distribution of most short-chain acyl-CoAs remain largely unexplored^133,138^.

The temporal and spatial regulation of acyl-CoAs in different cellular compartments, for example, the nucleus and mitochondria, provides an additional layer of control over acylations^138,139^. For instance, during nutrient deprivation or hypoxia, nuclear acetyl-CoA synthetase 2 (ACSS2) and ATP-citrate synthase (ACLY) ensure the supply of acetyl-CoA, which is generated from acetate and citrate, respectively, for histone acetylation^135,140^ (Fig. 4). Therefore, cells can ensure the maintenance of epigenetic modifications even following metabolic disruptions in the cytoplasm or mitochondria. Likewise, the dynamic regulation of nuclear acyl-CoAs also functions as a metabolic sensing mechanism, linking fluctuations in nuclear acyl-CoA levels directly to changes in gene expression patterns^138,139^. This mechanism enables cells to swiftly reprogram gene expression in response to nutrient availability, external signals or energy status.

### GTPSCS produces l-lactyl-CoA in the nucleus and promotes histone K_l-la_

We have recently identified GTP-specific succinyl-CoA synthetase (GTPSCS) as an l-lactyl-CoA synthetase in the nucleus^79^. In mammals, succinyl-CoA synthetase (SCS) functions as a mitochondrial enzyme existing in two complexes: ATPSCS and GTPSCS^141,142^. These two heterodimers comprise a common subunit, SUCLG1 (G1 subunit), which pairs with either SUCLA2 (A2 subunit of ATPSCS) or SUCLG2 (G2 subunit of GTPSCS), thereby forming two distinct isoforms with different cofactor preferences and metabolic roles. GTPSCS is highly expressed in the kidney and liver, producing ketone bodies and porphyrin^143,144^, whereas ATPSCS primarily functions in the tricarboxylic acid cycle, converting succinyl-CoA to succinate for ATP production^142,145^. We have demonstrated that GTPSCS robustly generates l-lactyl-CoA both in vitro and in vivo^79^.

**Fig. 5 |:**
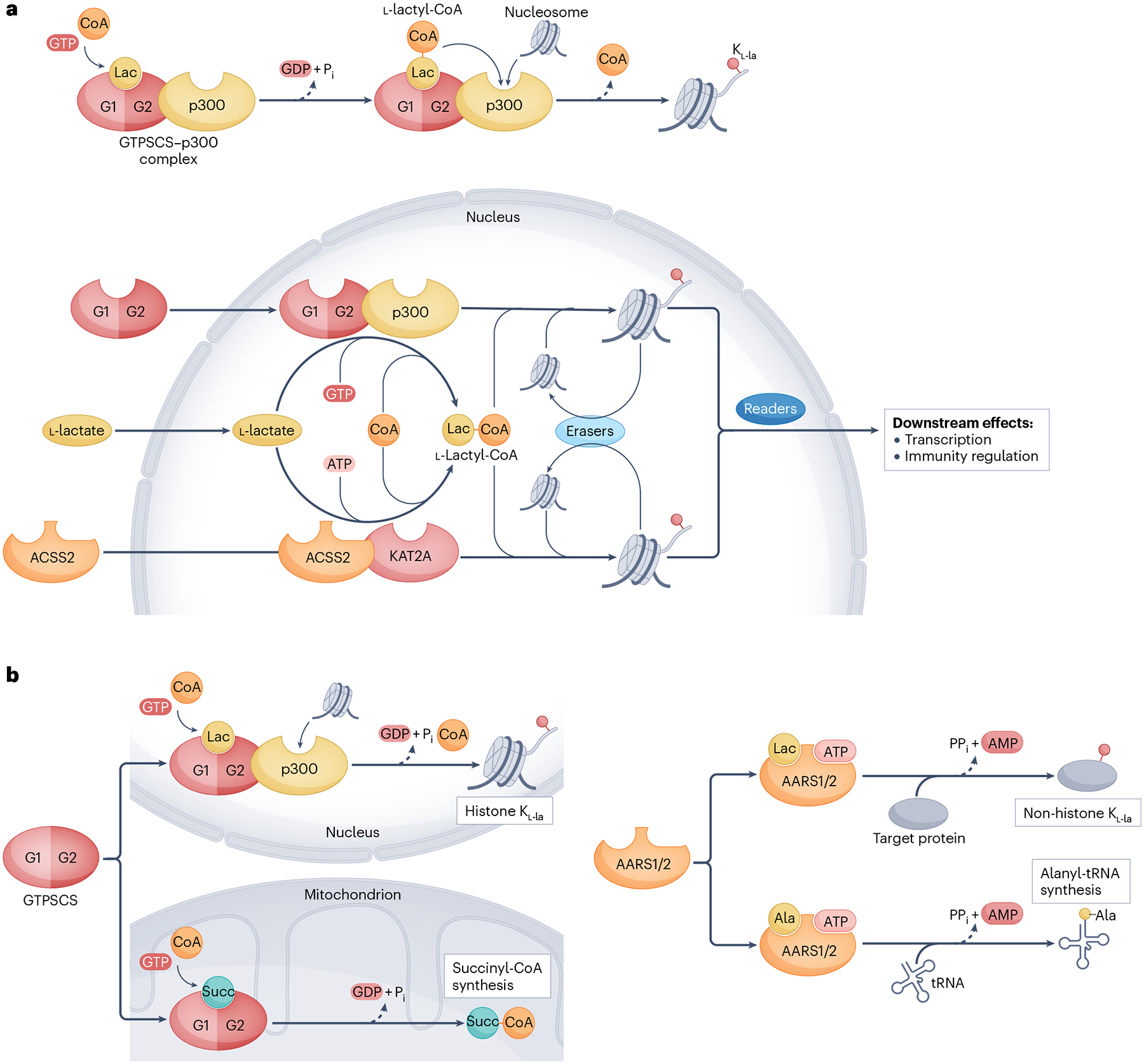
Histone K_l-la_ pathways. **a**, An overview of the two Lys l-lactylation (K_l-la_) pathways mediated by GTP-specific succinyl-CoA synthetase (GTPSCS) and acetyl-CoA synthetase 2 (ACSS2). Both can translocate into the nucleus and catalyse the formation of l-lactyl-CoA from l-lactate, coenzyme A (CoA) and, respectively, GTP or ATP. In the nucleus, GTPSCS and ACSS2 form complexes with p300 and Lys acetyltransferase 2A (KAT2A), respectively, to mediate histone K_l-la_ through an l-lactyl-CoA-dependent mechanism. Histone K_l-la_ can be removed by eraser enzymes such as histone deacetylases (HDACs) and sirtuins (SIRTs). **b**, The compartmentalized activities of GTPSCS and alanyl-tRNA synthetase 1 (AARS1) and AARS2 (AARS1/2). In mitochondria, GTPSCS catalyses the reversible conversion of succinate (Succ) to succinyl-CoA. Following translocation to the nucleus, GTPSCS synthesizes l-lactyl-CoA from l-lactate (Lac) and coenzyme A for histone K_l-la_. By contrast, the spatial regulation of AARS1 and AARS2, particularly in the nucleus, remains less defined. These enzymes mediate K_l-la_ through an alternative, l-lactyl-CoA-independent pathway that bypasses canonical lactyltransferases. Moreover, the canonical enzymatic activity of AARS1 drives alanyl-tRNA synthesis in the cytoplasm, whereas AARS2 fulfils this role in mitochondria. G1, G1 subunit in GTPSCS; Pi, phosphate; PPi, pyrophosphate.

In the nucleus, GTPSCS forms a functional lactyltransferase complex with p300 (Fig. 5a,b) to generate l-lactyl-CoA and subsequently transfer the lactyl group to histone Lys residues. GTPSCS nuclear translocation and l-lactyl-CoA production hinge on a nuclear localization signal in the G1 subunit^79^, and a Lys-to-Arg mutation in this region abolished its nuclear localization, suggesting that PTMs of GTPSCS regulate its nuclear function. The interaction between GTPSCS and p300 is crucial for histone K_l-la_. Disruption of the GTPSCS–p300 interaction interface abolishes histone K_l-la_^79^. By contrast, GTPSCS has little impact on the synthesis of nuclear succinyl-CoA and the regulation of histone succinylation^79^. The evidence supports a model whereby the nuclear-synthesized l-lactyl-CoA is rapidly used in situ by p300 for histone K_l-la_, potentially through substrate channelling^146^. Substrate channelling has been observed in several enzymes and multienzyme complexes, and in some cases, the architecture of the ‘tunnel’ that facilitates movement of a product from one enzyme to the active site of another has been mapped^146^. The structural basis of such a tunnel at the GTPSCS–p300 interface remains to be elucidated. Alternatively, a high local concentration of l-lactyl-CoA, combined with its rapid consumption by p300, may effectively drive histone K_l-la_, even in the absence of a dedicated channelling mechanism. Because p300 has robust activity towards l-lactyl-CoA and negligible activity towards succinyl-CoA, the l-lactyl-CoA generated by GTPSCS can be efficiently transferred to and used in situ by p300 for histone K_l-la_, thereby driving the enzymatic reaction towards the synthesis of l-lactyl-CoA.

l-lactyl-CoA is usually at lower concentrations than acetyl-CoA when whole-cell metabolites are analysed^147^. Nevertheless, the efficiency of enzymatic K_l-la_ reaction is dictated by local rather than by global cellular concentrations. Similar mechanisms exist in many other cases. For instance, KAT2A has strong succinyltransferase activity when binding to α-KGDH (an enzyme that catalyses the synthesis of succinyl-CoA), despite the low levels of nuclear succinyl-CoA^148^. The KAT2A–α-KGDH complex enables the sequential reaction of histone succinylation. Similarly, phase separation can concentrate enzymes and metabolites into confined spaces, thereby enhancing local substrate availability and enabling otherwise inefficient reactions^149,150^. In the case of K_l-la_, we propose that the GTPSCS–p300-mediated sequential reaction can overcome the relatively low affinity of GTPSCS for l-lactate and facilitate histone K_l-la_ despite low concentration of l-lactyl-CoA. This hypothesis is supported by the evidence that disrupting the GTPSCS–p300 complex eliminates GTPSCS-mediated histone K_l-la_^79^.

Histone K_l-la_ regulation by GTPSCS is crucial for gene transcription. Specifically, GTPSCS-regulated H3K18la supports the expression of tumour-promoting genes such as *GDF15*, which drives the growth of glioma tumours^79^. This effect is largely abolished by SUCLG2 depletion or mutation, and re-expression of GDF15 restored the inhibition of tumour cell proliferation caused by GTPSCS–p300–H3K18la inactivation^79^. The regulation of histone K_l-la_ by GTPSCS exemplifies how compartmentalized enzymatic activity driven by local substrate availability can drive specific epigenetic outcomes with physiological relevance in tumour biology.

In addition to GTPSCS, ACSS2 was recently reported to be another l-lactyl-CoA synthetase, working in conjunction with KAT2A as a histone lactyltransferase^82^. Upon activation of the epidermal growth factor receptor (EGFR) pathway, ERK phosphorylates ACSS2 at Ser267, prompting its translocation into the nucleus. In the nucleus, ACSS2 interacts with LDHA and KAT2A to form the LDHA–ACSS2–KAT2A complex (Fig. 5a). In this assembly, LDHA generates l-lactate, followed by generation of l-lactyl-CoA by ACSS2 and histone H3 K_l-la_ catalysed by KAT2A^82^.

The GTPSCS–p300 and ACSS2–KAT2A pathways operate through distinct mechanisms. ACSS2 is activated by EGFR–ERK signalling, whereas GTPSCS relies on a nuclear localization sequence and acetylation at its p300-interaction interface. These differences suggest that these two pathways might function in specific cellular contexts or complement one another to sustain histone K_l-la_ levels. Despite these differences, these two K_l-la_ pathways share key similarities: both involve an l-lactyl-CoA synthetase working in tandem with a lactyltransferase, ensuring the localized production and usage of l-lactyl-CoA for precise histone K_l-la_. Disruption of the two complexes has a substantial impact on histone K_l-la_ levels. These lines of evidence clearly indicate that lactyltransferase-dependent pathways are a major regulator of histone K_l-la_.

### The specificity of histone K_l-la_

Histone K_l-la_ shares writers and erasers with acetylation and other acylations, raising the important consideration of the specificity of histone K_l-la_. The specificity and output of histone acylations can theoretically be determined by any of the four classes of molecules: writers, erasers, readers and molecules involved in cofactor biosynthesis.

The substrate specificity of acyltransferases towards acyl-CoAs is shaped by factors such as acyl-CoA chain length, PTM-induced conformational changes and by interacting partners (for example, proteins and metabolites). One notable example is p300, which exhibits broad activity towards various short-chain acyl-CoAs, such as acetyl-CoA, propionyl-CoA, butyryl-CoA, crotonyl-CoA and l-lactyl-CoA but not towards long-chain acyl-CoAs, such as malonyl-CoA, succinyl-CoA and glutaryl-CoA^108^. Further proteomics and biochemical analyses suggested that p300 can selectively use distinct acyl-CoA variants, thereby defining acylation profiles that differ, for example, between acetylation and 2-hydroxyisobutyryaltion^151^. Collectively, these findings indicate that the specificity of p300 is shaped by acyl-CoA structure, substrate context, PTMs and protein-complex assembly.

The association between p300 and GTPSCS enables p300 to use the locally generated l-lactyl-CoA for histone K_l-la_, thus highlighting the specificity of histone K_l-la_ through l-lactyl-CoA synthesis. GTPSCS can use both succinate and l-lactate as substrates and was shown to regulate nuclear l-lactyl-CoA and histone K_l-la_ but not nuclear succinyl-CoA and Lys succinylation^79^. It is notable that succinyl-CoA is far more efficient at non-enzymatic acylation of Lys residues compared with most other acyl-CoAs, because succinyl-CoA can spontaneously undergo a cyclization to an anhydride, which is highly reactive^152^. Therefore, GTPSCS does not favour Lys succinylation of histones compared with l-lactylation. The preferential production of l-lactyl-CoA over succinyl-CoA by GTPSCS is driven by substrate availability and enzymatic activity. In cultured U87 glioma cells, succinate exists at substantially lower concentrations (0.029 mM) compared with l-lactate (1.68–5.40 mM)^79^. When supplied with succinate and l-lactate at these concentrations, GTPSCS produces up to sixfold more l-lactyl-CoA than succinyl-CoA in vitro, suggesting that l-lactyl-CoA production is more favourable in cellular contexts^79^. This substrate-driven specificity can be further amplified in some cellular conditions, in which l-lactate concentration can reach up to 10–40 mM (refs. 29,91–94). The preference of l-lactyl-CoA by p300, in combination with the enzymatic specificity of GTPSCS towards production of l-lactyl-CoA over succinyl-CoA and acetyl-CoA in the nucleus, drives histone K_l-la_ rather than K_ac_ or K_succ_^108^.

### Enzyme-catalysed versus non-enzymatic K_l-la_

Acyl-CoAs can non-enzymatically react with Lys ε-amino groups, raising the possibility that histone K_l-la_ can arise through passive chemistry. Non-enzymatic acylation reactions are generally promiscuous and lack substrate specificity, but their efficiency can be influenced by local acyl-CoA availability and pH^153–155^. For example, non-enzymatic acetylation is prevalent in mitochondria, where acetyl-CoA is abundant and the pH (7.9–8) is higher than in the cytosol and nucleus (~7.2)^154^. K_l-la_ is primarily an enzyme-catalysed modification, as supported by converging evidence from in vitro biochemical assays, cell-based experiments and proteomics studies. First, in vitro assays have shown that several histone lactyltransferases, such as p300, CBP, KAT2A, KAT5, KAT7 and KAT8, can directly catalyse K_l-la_ using l-lactyl-CoA^28,38,65,67,68^. Second, inhibition of these lactyltransferases reduces K_l-la_ levels across diverse cell types, confirming their intracellular activities^28,34,38,53,65,67,68,78,84,109–111^. Consistent with these findings, the localization and function of the l-lactyl-CoA synthetase GTPSCS are crucial for histone K_l-la_ in glioma cell lines. GTPSCS depletion markedly downregulated (~50%) histone K_l-la_, and disruption of its nuclear localization or interaction with p300 dramatically reduced (~50%) histone K_l-la_ levels in some cultured cells^79^. Finally, histone K_l-la_ marks exhibit specific chromatin distributions, in contrast to random, non-enzymatic modification^28,36,39,53,65,68,80^. Together, these findings provide strong evidence that histone K_l-la_ is an enzyme-mediated process rather than a by-product of passive chemical reactivity.

## Stoichiometry of K_l-la_

Methods for measuring PTM stoichiometry have yielded divergent results^153,156–158^. Accurate measurement of the stoichiometry of a post-translationally modified site requires the use of isotopically labelled synthetic peptides, which is expensive and time consuming. Histone K_l-la_ can be detected by HPLC with tandem mass spectrometry analysis even without enrichment (Y.Z., unpublished results), suggesting that it is not present in extremely low levels. Despite extensive studies in the past several years, no study has systematically defined the stoichiometry of histone K_l-la_.

Previous studies show that low stoichiometry of histone modifications is not correlated with their functional significance. For example, K_ac_ exhibits a wide range of stoichiometry in human cells, varying from over 10% to less than 0.01%^153,156,157^. Despite their relatively low abundance, H3K9ac and H3K27ac are considered as marks of active chromatin and are associated with increased gene expression^104,159^. Likewise, trimethylation of H3K4 (H3K4me3) and H3K27me3 are typically present in extremely low quantities and are barely detectable by mass spectrometry in certain cell types^160–162^. Nevertheless, they have crucial roles in the formation of so-called chromatin ‘bivalent domains’, which mark key developmental genes and regulate transcription in embryonic stem cells^163^. Early studies have demonstrated the existence of alternating patterns of histone acetylation and longer-chain acylations at a subset of nucleosomes^164,165^, which overlap with transcription start sites of active genes^166,167^. It was proposed that differential affinity of bromodomain proteins, in particular BRD4 and BRDT, towards Lys acylations (for example, lower affinity for butyrylation and crotonylation), may contribute to transcriptional reprogramming in response to elevated levels of longer-chain histone acylations (irrespective of their nature) compared with acetylation^167,168^. These findings also suggest that, in the context of histone binding by BRD4 (and BET factors in general), histone acetylation (that is, H4K5ac) and the sum of other longer-chain acylations compete for occupancy at the same Lys residues.

Importantly, the balance between acetylation and other acylations is highly dynamic, allowing reader proteins to bind and dissociate from histone modifications in response to metabolic fluctuations. This balance enables fine tuning of the chromatin environment and precise control of gene expression, without global alteration in acylation stoichiometry. The disconnection between low global stoichiometry and high functionality of histone modifications suggests that even modifications present at low global levels can reach high local concentrations and exert considerable biological effects. In addition, the recent discovery of ATP-dependent carriers of acetyl-CoA and longer-chain acyl-CoAs suggests that these metabolites are locally delivered to specific chromatin regions, promoting site-specific histone acylation without altering global modification levels. In the case of K_l-la_, the discovery of the p300–GTPSCS complex is in agreement with this notion, and localized histone K_l-la_ may regulate transcription and downstream effects irrespective of its overall stoichiometry. Moreover, the role of K_ac_ as a reversible acetate reservoir suggests that some modifications may exist at low global levels mainly because they are metabolically tuned and locally mobilized in response to cellular demands^169–171^, further underscoring that stoichiometry alone does not predict function.

In addition, low-stoichiometry PTMs may also have important roles through other means. Inhibition of protein function often necessitates a relatively high stoichiometry of the inhibiting PTM. However, when the basal activity of a protein is low, gain-of-function may occur with much lower stoichiometry^156^. Such examples have been reported for K_l-la_ substrate proteins whose functions are regulated by K_l-la_-dependent protein–protein interactions, protein translocation or conformational changes^36,39,84^. These mechanisms exist for other PTMs as well^3,152,172,173^.

## An l-lactyl-CoA-independent l-lactylation pathway

Since the initial discovery of K_l-la_ and p300 as a lactyltransferase in 2019, alanyl-tRNA synthetase 1 (AARS1; also known as alanine-tRNA ligase, cytoplasmic) and AARS2 (also known as alanine-tRNA ligase, mitochondrial) were recently reported to mediate K_l-la_ in an l-lactyl-CoA-independent manner^174,175^ (Fig. 5b). Although traditionally known to synthesize aminoacyl-tRNA, AARS1 and AARS2 (AARS1/2) were reported to use l-lactate as a precursor for l-lactyl-AMP, thus bypassing the need for l-lactyl-CoA and KAT-like lactyltransferases^174,175^. AARS1 can catalyse the lactylation reaction on various substrate proteins and thus regulate their functions^174–176^.

The two K_l-la_ pathways — l-lactyl-CoA-dependent and l-lactyl-CoA-independent — exhibit notable biochemical differences. The l-lactyl-CoA-dependent pathway relies on an enzyme synthesizing l-lactyl-CoA. GTPSCS has a calculated *K*_m_ value for l-lactate of 15 mM, which falls within the range of physiological l-lactate tissue concentrations (0.5–40 mM)^29,91–94^. ACSS2 has a *K*_m_ value less than 1 mM, demonstrating high binding affinity^82^. The sequential reaction performed either by GTPSCS–p300 or ACSS2–KAT2A enables efficient use of the locally generated l-lactyl-CoA for K_l-la_, thereby accelerating the reaction. AARS1 demonstrates a higher *K*_m_ value for l-lactate (36 mM), suggesting a lower apparent affinity for its substrate. AARS2 has a lower *K*_m_ (5 mM) and higher catalytic efficiency^174,175^.

Each of the four enzymes — GTPSCS, ACSS2 and AARS1/2 — has dual enzymatic activities (Fig. 5a,b). Thus, separating the two activities is fundamental for distinguishing their K_l-la_-mediated function from their known canonical functions. GTPSCS predominantly regulates the production of l-lactyl-CoA but not succinyl-CoA in the nucleus^79^. The GTPSCS-mediated K_l-la_ nuclear functions can be easily differentiated from its mitochondrial functions by mutation of either its nuclear localization signals or its p300-interacting residue. Likewise, depletion and overexpression of ACSS2 altered the nuclear levels of l-lactyl-CoA more than those of acetyl-CoA following EGF treatment^82^. The distinction between l-lactyl-CoA and acetyl-CoA can be achieved by Arg533 of KAT2A, and a R533A mutation selectively reduced histone H3 K_l-la_ but not K_ac_^82^. By contrast, AARS1 has a strong preference for Ala (*K*_m_ = 80 μM) over l-lactate (*K*_m_ = 36 mM), and it has a stronger binding affinity to Ala (*K*_d_ = 0.45 μM) over l-lactate (*K*_d_ = 35 μM)^174,175^. Given that intracellular Ala concentrations (~0.5 mM) are comparable with those of l-lactate in normal conditions^177,178^, Ala is probably the predominant substrate and can inhibit the production of l-lactyl-AMP by AARS1/2. The crucial role of alanyl-tRNA in translation and the Ala preference of AARS1/2 raise an important question: are the consequences of AARS1/2 deficiency driven by reduction in K_l-la_ levels or by disruption of alanyl-tRNA generation and thus protein synthesis?

## Proteomics studies for identifying K_l-la_ substrates

Dozens of proteomics studies, including both l-lactyl-CoA-dependent and l-lactyl-CoA-independent pathways, were carried out to identify K_l-la_ substrates^55–64,179,180^. A few studies have identified lactyltransferase-dependent K_l-la_ substrates^28,65–67^. The identified K_l-la_ substrates are localized not only in the nucleus but also in other organelles, including the mitochondria and cytosol, suggesting that multiple mechanisms exist for K_l-la_ modification of substrates. These studies also infer roles of K_l-la_ substrates in diverse processes, including inflammation, chromatin assembly and mRNA processing. Furthermore, studies of l-lactyl-CoA-dependent, lactyltransferase-mediated substrates demonstrated a crucial role of this pathway in diverse physiological conditions, including macrophage polarization, tumour growth, osteoblast differentiation, polymicrobial sepsis, neovascularization, atherosclerosis, embryonic development and myocardial infarction^28,36,39,67,78–80,84,109–111^.

In addition, histones have been reported as targets of multiple lactyltransferases, including p300, CBP, KAT7, KAT2A and KAT5 (refs. 28,65,68,79,82,111), suggesting that AARS1/2 are not the primary drivers of histone K_l-la_. However, no studies have systematically assessed the regulation of histone K_l-la_ levels by the AARS1/2-mediated, l-lactyl-CoA-independent pathway. In fact, K_l-la_ sites on histone proteins were far less affected by AARS1/2 activity compared with non-histone proteins^174^ (Fig. 5b). Proteomics quantification of AARS1/2-mediated K_l-la_ substrates is complicated by the fact that AARS1/2 are also important for translation and that genetic manipulation of AARS1/2 can change protein abundances. To identify AARS1/2-mediated K_l-la_ substrates, quantitative proteomics analyses should be conducted by normalization to protein abundances, which ideally requires a specific mutant with abolished activity in l-lactylation but not in translation. Unfortunately, such studies have not been carried out yet.

## Genetic dissection of lactyltransferase functions

In vitro biochemical studies offer a starting point for investigating the function of a K_l-la_ substrate; however, mutagenesis and genetic studies are crucial for establishing causative relationships. Through targeted point mutations, the activity of GTPSCS towards synthesizing nuclear l-lactyl-CoA can be specifically differentiated from its mitochondrial function of reversing succinyl-CoA synthesis. These mutations in GTPSCS alter its nuclear localization signal or key residues at its interaction interface with p300, enabling selective disruption of histone K_l-la_ without affecting other known functions^79^. Genetic experiments have further supported the role of GTPSCS in tumour growth and macrophage functions^79^. Specifically, SUCLG2 knockout in glioma cells suppresses tumour growth, a reduction linked to decreased histone K_l-la_, such as H3K18la, and altered expression of the tumour-promoting gene *GDF15* (ref. 79).

Likewise, the l-lactyl-CoA synthesis function of ACSS2 is regulated by Ser267 phosphorylation, nuclear translocation and interaction with KAT2A. Genetic studies of ACSS2–KAT2A demonstrated that disrupting the interaction between ACSS2 and KAT2A effectively reduced tumour growth without altering EGF-induced histone acetylation levels and other EGF-mediated changes^82^. Similarly, mutating the ERK2-targeted phosphorylation site of ACSS2 also affected tumour growth and immune cell infiltration^82^.

Studying the function of AARS1/2-mediated K_l-la_ is more complicated, because these enzymes are indispensable for protein synthesis^181,182^. Thus, disruption of their activity inevitably affects their primary function. Although proteomic analyses have been conducted in AARS1-knockout and AARS2-knockout cells, the quantification of K_l-la_ was not normalized to protein abundance, leaving it unclear whether the observed functional consequences stem from changes in protein synthesis or K_l-la_^174,175^. To address this problem, a genetic screen would be instrumental in identifying AARS1/2 mutations that can selectively affect the K_l-la_ or alanyl-tRNA synthesis activities to establish the causal role of AARS1/2-mediated K_l-la_.

## Concluding remarks and future perspective

Advances in the past two decades have revealed many Lys acylation pathways, considerably expanding our understanding of histone modifications. Extensive studies have clearly demonstrated that histone Lys short-chain acylations have pivotal roles in regulating gene expression and in other, transcription-independent functions. Importantly, Lys acylations are associated with multiple genetic diseases characterized by accumulation of short-chain lipids and elevated levels of Lys acylations. These diseases include short-chain acyl-CoA dehydrogenase deficiency^183,184^, malonyl-CoA decarboxylase deficiency^185,186^, deficiency of glutaryl-CoA dehydrogenase^187–189^ and deficiency of succinyl-CoA synthetase^173,190–193^. Lys acylations, including but not limited to propionylation^194^, butyrylation^195^, malonylation^196^, succinylation^197,198^, β-hydroxybutyrylation^199^ and glutarylation^189^, are also implicated in pathological conditions such as cancer, metabolic disorders, diabetes and immune dysfunction.

The elevated levels of l-lactate in diverse pathophysiological conditions^200–203^, for example, in innate immunity responses, tumorigenesis, hypoxia and the Warburg effect, stimulate K_l-la_. Mounting evidence suggests that K_l-la_ contributes to the regulation of gene expression through core histones and many non-histone proteins^28,36,39,68,80^. Given the complexity of K_l-la_ pathways, we believe that the research community would benefit from taking the following measures in future studies. It is clear that K_l-la_, K_d-la_ and K_ce_ are three distinct PTMs. Accordingly, to avoid confusion, it is necessary to explicitly specify which Lys isomer is under investigation. Similarly, lactate and lactyl-CoA should be labelled as either the l-isoform or d-isoform in experimental contexts. To unambiguously define the function of the K_l-la_ pathways, we suggest conducting control experiments to assess the effects of K_l-la_ levels through either l-lactyl-CoA-dependent or l-lactyl-CoA-independent pathways. To this end, genetic manipulation of the relevant enzymes in both pathways is needed to define the specific relevant pathway and K_l-la_ substrates. Furthermore, the multiple functions of K_l-la_ regulating enzymes should be analysed with appropriate mutants.

Further investigations of K_l-la_ should focus on three key directions. First, many proteomics analyses of acylations were conducted in cell culture systems. To better understand the physiological functions of K_l-la_, it is crucial to perform comprehensive quantification across tissues and disease states. In addition, similar quantitative proteomics studies are needed to identify the specific K_l-la_ targets of GTPSCS–p300, ACSS2–KAT2A, AARS1 and AARS2 and of other writer and eraser enzymes. Second, disentangling the dual functions of GTPSCS, ACSS2 and AARS1/2 is essential for future functional studies. Function-selective mutants are crucial for dissecting the dual functions of these enzymes and establish causative relationships. Third, the regulation of K_l-la_ remains to be better elucidated. A complete picture of K_l-la_ requires identifying additional writer, eraser and reader proteins, which are either shared with other acylations or specific to K_l-la_. Future studies along these lines would establish a robust foundation for investigating the functions of K_l-la_ on both core histones and non-histone proteins. Finally, l-lactate is closely associated with pathologies such as cancer. Accordingly, l-lactate-stimulated K_l-la_ could provide an avenue for therapeutic intervention. Chemical inhibitors of regulatory enzymes specific to K_l-la_ but not to other acylaitons (for example, acetylation), will probably be less toxic than inhibitors of HATs and HDACs owing to their greater specificity.

## Supplementary Material

Supplementary table

**Supplementary information** The online version contains supplementary material available at https://doi.org/10.1038/s41580-025-00876-7.

## Glossary

ε-amine: The amino group at the side chain of Lys.
Bromodomain: A protein domain that specifically binds to acetylated lysine residues of histones; often found in chromatin-associated proteins and acetyltransferases.
Chiral centre: A three-dimensional configuration of atoms around a carbon atom that leads to the formation of stereoisomers such as Lys l-lactylation and Lys d-lactylation.
Modified single amino acid analysis: A method involving chemical derivatization to differentiate stereoisomers such as Lys l-lactylation and Lys d-lactylation.
PHD finger domain: A zinc-binding domain that recognizes methylated lysine residues of histones.
Substrate channelling: Direct transfer of intermediates between enzymes without diffusion into the surrounding medium; proposed for GTP-specific succinyl-CoA synthetase (GTPSCS)–p300-mediated Lys l-lactylation.
YEATS domain: An acetylation-binding domain with a unique structural pocket that accommodates a wide range of acyl modifications.
