# Supplementary material for: Biochemistry and regulation of histone lysine l-lactylation
Source: Nat Rev Mol Cell Biol. Author manuscript; Available in PMC 2026 Feb 19. (PMC12920031; doi:10.1038/s41580-025-00876-7)
Supplement: Supplementary table [file NIHMS2136976-supplement-Supplementary_table.pdf]

Table 1: Structures of recently identified Lys acylations

Name	Structure	Mass shift (Da)	Year of discovery
Benzoylation (K_{bz})	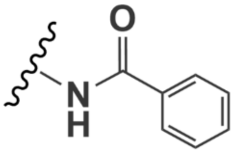	+104.027	(Huang et al. 2018)
L-Lactylation ($K_{L\text{-}la}$)	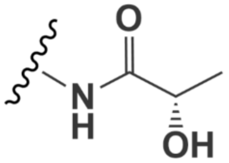	+72.021	(Zhang et al. 2019)
Methacrylation (K_{mea})	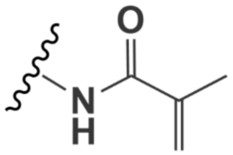	+68.023	(Delaney et al. 2021)
Isobutyrylation (K_{ibu})	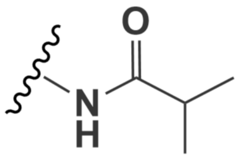	+ 70.041	(Zhu et al. 2021)
Isonicotinylation (K_{inic})	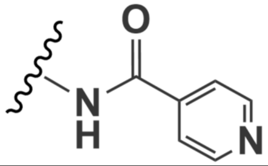	+105.022	(Jiang et al. 2021)
Acetyl-methylation (K_{acme})	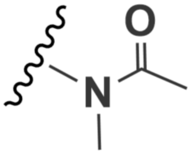	+56.026	(Lu-Culligan et al. 2023)
Itaconylation (K_{ita})	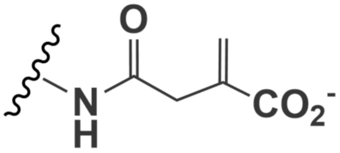	+112.016	(Liu et al. 2023)
Acetoacetylation (K_{acac})	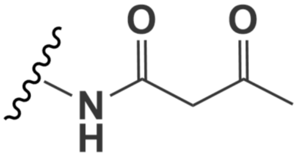	+84.021	(Gao et al. 2023)

Delaney, K., M. Tan, Z. Zhu, J. Gao, L. Dai, S. Kim, J. Ding, M. He, L. Halabelian, L. Yang, P. Nagarajan, M. R. Parthun, S. Lee, S. Khochbin, Y. G. Zheng, and Y. Zhao. 2021. 'Histone lysine methacrylation is a dynamic post-translational modification regulated by HAT1 and SIRT2', *Cell Discov*, 7: 122.

Gao, Y., X. Sheng, D. Tan, S. Kim, S. Choi, S. Paudel, T. Lee, C. Yan, M. Tan, K. M. Kim, S. S. Cho, S. H. Ki, H. Huang, Y. Zhao, and S. Lee. 2023. 'Identification of Histone Lysine Acetoacetylation as a Dynamic Post-Translational Modification Regulated by HBO1', *Adv Sci (Weinh)*, 10: e2300032.

Huang, H., D. Zhang, Y. Wang, M. Perez-Neut, Z. Han, Y. G. Zheng, Q. Hao, and Y. Zhao. 2018. 'Lysine benzoylation is a histone mark regulated by SIRT2', *Nat Commun*, 9: 3374.

Jiang, Y., Y. Li, C. Liu, L. Zhang, D. Lv, Y. Weng, Z. Cheng, X. Chen, J. Zhan, and H. Zhang. 2021. 'Isonicotinylation is a histone mark induced by the anti-tuberculosis first-line drug isoniazid', *Nat Commun*, 12: 5548.

Liu, D., W. Xiao, H. Li, Y. Zhang, S. Yuan, C. Li, S. Dong, and C. Wang. 2023. 'Discovery of Itaconate-Mediated Lysine Acylation', *J Am Chem Soc*, 145: 12673-81.

Lu-Culligan, W. J., L. J. Connor, Y. Xie, B. E. Ekundayo, B. T. Rose, M. Machyna, A. P. Pintado-Urbanc, J. T. Zimmer, I. W. Vock, N. V. Bhanu, M. C. King, B. A. Garcia, F. Bleichert, and M. D. Simon. 2023. 'Acetyl-methyllysine marks chromatin at active transcription start sites', *Nature*, 622: 173-79.

Zhang, D., Z. Tang, H. Huang, G. Zhou, C. Cui, Y. Weng, W. Liu, S. Kim, S. Lee, M. Perez-Neut, J. Ding, D. Czyz, R. Hu, Z. Ye, M. He, Y. G. Zheng, H. A. Shuman, L. Dai, B. Ren, R. G. Roeder, L. Becker, and Y. Zhao. 2019. 'Metabolic regulation of gene expression by histone lactylation', *Nature*, 574: 575-80.

Zhu, Z., Z. Han, L. Halabelian, X. Yang, J. Ding, N. Zhang, L. Ngo, J. Song, H. Zeng, M. He, Y. Zhao, C. H. Arrowsmith, M. Luo, M. G. Bartlett, and Y. G. Zheng. 2021. 'Identification of lysine isobutyrylation as a new histone modification mark', *Nucleic Acids Res*, 49: 177-89.
